# The Influence of Multiple Frailties on Institutionalization and Mortality in Community-Living Older Adults

**DOI:** 10.1093/geroni/igaa057.825

**Published:** 2020-12-16

**Authors:** Yunhwan Lee

**Affiliations:** Ajou University School of Medicine, Suwon, Republic of Korea

## Abstract

Frailty is a hallmark of accelerated aging, predisposing the older person to increased vulnerability to adverse health outcomes. Physical frailty is closely linked to other health dimensions, such as cognitive, psychological, and social functions. This study aims to examine the influence of multiple dimensions of frailty in predicting institutionalization and mortality. A nationally representative sample of Koreans 65 years or older from the Living Profiles of Older People Survey in 2008 was followed up for three years (n = 11,265). Physical frailty was defined as being prefrail or frail using the Fried phenotype model. Those with cognitive impairment, depressive symptoms, and social vulnerabilities, in addition to physical frailty, were considered to have cognitive frailty, psychological frailty, and social frailty, respectively. The proportional hazards model was used to analyze the risk of institutionalization and mortality by the total number and different combinations of frailties, adjusting for covariates. More than half (50.1%) of the participants exhibited multiple frailties, with 8.1% concurrently displaying frailty in all four domains (mixed frailty). The risk of adverse outcomes was elevated with a higher number of frailties, with hazard ratios of 2.59 (95% confidence interval [CI]: 1.52, 4.42) for institutionalization and 3.40 (95% CI: 2.50, 4.63) for mortality among those presenting mixed frailty. Whereas psychological frailty demonstrated a stronger predictive ability of mortality than institutionalization, the reverse was observed for social frailty. Multiple frailties are prevalent in late life. Acquiring more frailties raises the risk of adverse outcomes, with varying effects according to multidimensional frailty profiles.

